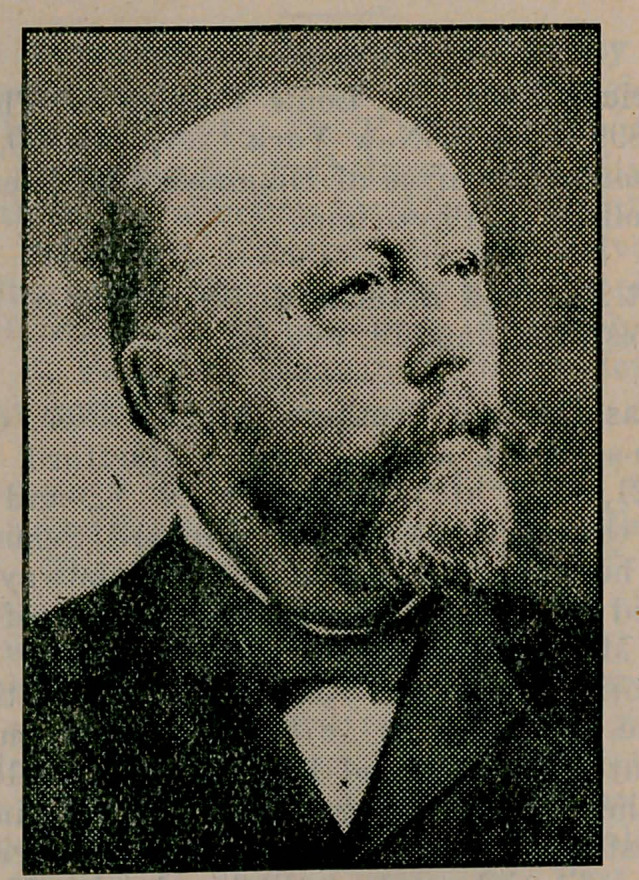# Dr. Conrad Diehl

**Published:** 1918-04

**Authors:** 


**Dr. Conrad Diehl** died in Buffalo, February 20, age 75. He was graduated from the University of Buffalo in 1866. He left shortly afterwards for courses in the Universities of Gottingen and Berlin. He served as Coroner of Erie Co. in 1868,

Mayor of Buffalo in 1897-1901, and was the chairman of the first Board of School Examiners in 1896. From 1870 to 1878 he served as Major in the Medical Corps of the 65th Regiment. During Dr. Diehl's whole life, he had lived in but two houses, situated a few blocks from each other.

At a special meeting of the Medical and Surgical Staff of the Buffalo General Hospital a committee was appointed by Dr. James E. King, President of the Staff, consisting of Dr.

Henry R. Hopkins, chairman; Dr. Norman L. Burnham and Dr. Allen A. Jones, to draft a suitable memorial upon the death of Dr. Conrad Diehl.

Our late lamented confrere, Dr. Conrad Diehl, possessed many of the finest and best characteristics of the good, the worthy, the capable, and the efficient physician. He began his medical career well equipped and fortified by study and experience gained here and abroad. He early engaged in more or less public and institutional medicine. His sterling makeup, his probity, his ready and spontaneous kindliness, his professional abilities, quickly won him practice and standing in the community. His freedom from pretence and vanity won for him confidence and respect. His conscientious industry increased his practice, set him as dependable in the hearts of his large clientele, and made him much esteemed.

His popularity as a citizen was measured by his election as Mayor of the City of Buffalo, and he served honorably in that capacity in 1901, at the time of our beautiful Pan-American Exposition. In his public capacity he stood by and ministered to our late beloved President, William McKinley, whose sad and untimely death spread its gloom upon the city.

His enthusiastic, willing, and unstinted service to The Buffalo General Hospital are well known to many who survive him, and were appreciated by many who served with him and departed before him. The members of the Medical and Surgical Staff of the hospital, therefore, hereby testify to their deep sense of the splendid services rendered by their deceased and revered colleague; they keenly regret his loss; and they extend to his bereaved family their sincere and heartfelt sympathy.

## Figures and Tables

**Figure f1:**